# Detecting and accounting for multiple sources of positional variance in peak list registration analysis and spin system grouping

**DOI:** 10.1007/s10858-017-0126-5

**Published:** 2017-08-16

**Authors:** Andrey Smelter, Eric C. Rouchka, Hunter N. B. Moseley

**Affiliations:** 1 School of Interdisciplinary and Graduate Studies, University of Louisville, Louisville, KY 40202 USA; 2 Department of Computer Engineering and Computer Science, University of Louisville, Louisville, KY 40202 USA; 3 KBRIN Bioinformatics Core, University of Louisville, Louisville, KY 40202 USA; 4 Department of Molecular and Cellular Biochemistry, University of Kentucky, Lexington, KY 40356 USA; 5 Markey Cancer Center, University of Kentucky, Lexington, KY 40356 USA; 6 Center for Environmental and Systems Biochemistry, University of Kentucky, Lexington, KY 40356 USA; 7 Institute for Biomedical Informatics, University of Kentucky, Lexington, KY 40356 USA

**Keywords:** Spin system grouping, Variance-informed DBSCAN, Peak list registration and alignment analysis, Simulated peak list with variance, Nuclear magnetic resonance

## Abstract

Peak lists derived from nuclear magnetic resonance (NMR) spectra are commonly used as input data for a variety of computer assisted and automated analyses. These include automated protein resonance assignment and protein structure calculation software tools. Prior to these analyses, peak lists must be aligned to each other and sets of related peaks must be grouped based on common chemical shift dimensions. Even when programs can perform peak grouping, they require the user to provide uniform match tolerances or use default values. However, peak grouping is further complicated by multiple sources of variance in peak position limiting the effectiveness of grouping methods that utilize uniform match tolerances. In addition, no method currently exists for deriving peak positional variances from single peak lists for grouping peaks into spin systems, i.e. spin system grouping within a single peak list. Therefore, we developed a complementary pair of peak list registration analysis and spin system grouping algorithms designed to overcome these limitations. We have implemented these algorithms into an approach that can identify multiple dimension-specific positional variances that exist in a single peak list and group peaks from a single peak list into spin systems. The resulting software tools generate a variety of useful statistics on both a single peak list and pairwise peak list alignment, especially for quality assessment of peak list datasets. We used a range of low and high quality experimental solution NMR and solid-state NMR peak lists to assess performance of our registration analysis and grouping algorithms. Analyses show that an algorithm using a single iteration and uniform match tolerances approach is only able to recover from 50 to 80% of the spin systems due to the presence of multiple sources of variance. Our algorithm recovers additional spin systems by reevaluating match tolerances in multiple iterations. To facilitate evaluation of the algorithms, we developed a peak list simulator within our nmrstarlib package that generates user-defined assigned peak lists from a given BMRB entry or database of entries. In addition, over 100,000 simulated peak lists with one or two sources of variance were generated to evaluate the performance and robustness of these new registration analysis and peak grouping algorithms.

**Electronic supplementary material:**

The online version of this article (doi:10.1007/s10858-017-0126-5) contains supplementary material, which is available to authorized users.

## Introduction

One of the prerequisite analyses for protein structure determination is the assignment of chemical shifts to specific nuclei in a protein structure. During the assignment process, spin systems are mapped to individual amino acid residues in a protein sequence. In general, a spin system can be viewed as a group of nuclear spins that interact with each other in a magnetic field. In this study, we define a spin system as a collection of related resonances associated with specific atoms in a molecule that can be grouped within a single spectrum and across multiple spectra with common resonances. In the context of biopolymers such as proteins, spin systems often represent resonances associated with atoms within one, two, or even three bonded residues. Manual resonance assignment is tedious and can take a significant amount of time. Therefore, a variety of automated and semi-automated assignment programs have been developed to facilitate the protein resonance assignment process, specifically for solution (Shimotakahara et al. 1997; Moseley and Montelione 1999; Wang et al. 2005; Crippen et al. 2010; Schmidt and Güntert 2012; Niklasson et al. 2015; Guerry and Herrmann 2011) and solid-state NMR (Moseley et al. 2010; Tycko and Hu 2010; Schmidt et al. 2013; Nielsen et al. 2014). The process of automated resonance assignment typically involves several major steps: grouping peaks across peak lists into spin systems, classification of those spin systems by possible amino acid type, linking neighboring spin systems into segments, and then mapping those segments onto protein sequence.

### Lack of automated tools to determine match tolerances

One of the historical problems that has limited the use of automated and semi-automated protein resonance assignment tools along with other analyses of NMR peak lists is the requirement that users either specify uniform match tolerances typically for ^1^H and ^15^N resonances (for solution NMR) and ^15^N, and ^13^C resonances (for solid-state NMR) to perform spin systems grouping and linking, or rely on default uniform match tolerance values. Some programs even expect the user to provide spin systems instead of peak lists (Coggins and Zhou 2003). In essence, the user is left to determine which match tolerances should be used for their dataset. Restated, basic peak positional variance statistics that could be derived from the peak lists data are being required from the user, limiting the utility of these tools. Also, these same peak list statistics are useful for assessing the quality of peak lists, especially for downstream analyses (Baran et al. 2004; Moseley et al. 2001).

### Presence of multiple sources of variance

**Fig. 1:**
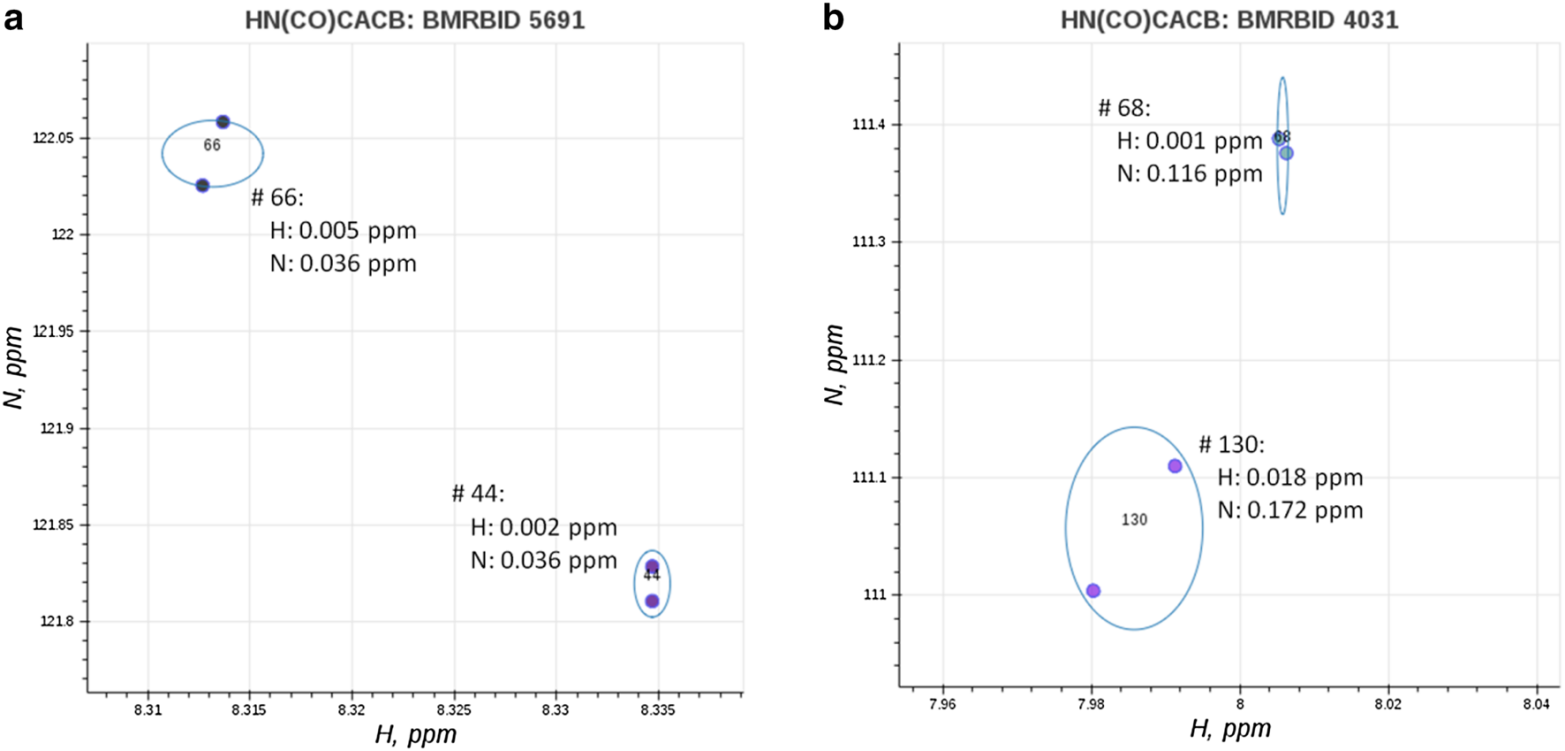
Zoomed-in visualization of spin systems taken from two experimental HN(CO)CACB peak lists that demonstrates the presence of multiple sources of variance within peak lists. The *dots* correspond to peak centers, two peaks form an individual spin system, ovals show the per-dimension variance (bivariance): **a** for the 30S ribosomal protein S28E from *P. horikoshii*, spin systems 44 and 66 show variance in the H dimension; **b** for pancreatic ribonuclease both spin systems 68 and 130 show variance in both H and N dimensions

Another problem that exists in experimental peak lists derived from both solution and solid-state NMR experiments is the presence of multiple variances in dimension-specific peak positions. In effect, there is a subset of peaks within a single peak list that have a smaller variance and can be grouped into spin systems using tighter match tolerance values, and a subset of peaks that have a larger variance in one or all dimensions that require larger match tolerance values for grouping into spin systems. On the one hand, using tighter tolerance values could result in failure to group peaks with larger variances, on the other hand using larger tolerance values could result in spin system overlap in peaks that have a smaller variance. This also limits the utility of uniform match tolerances for spin system grouping, linking and mapping algorithms. Figure 1 demonstrates the presence of peak groups (clusters) with multiple sources of variance in peak positions within experimental HN(CO)CACB peak lists. For the 30S ribosomal protein S28E from *Pyrococcus horikoshii* in Fig. 1a, the two visualized spin systems demonstrate different sources of variances in the amide ^1^H dimension. For the pancreatic ribonuclease in Fig. 1b, the visualized spin systems demonstrate multiple sources of variance in both amide ^1^H and ^15^N dimensions. These multiple sources of variances arise from an array of sample conditions, analytical conditions, experimental parameters, and spectral artefacts that can each contribute a different source of variation to a peak’s position, i.e. center.

AutoAssign, an automated resonance assignment software for solution NMR HN-based peak lists, was the first automated protein resonance assignment tool to provide the ability to register different peak lists, extract peak list quality statistics, and offset registration values necessary to align a set of peak lists against a specified reference peak list (Moseley et al. 2010; Monleón et al. 2002). In essence, the registration analysis algorithm finds the global offset values that necessary to apply to each peak within peak list of interest in order for it to match target peak list, i.e. it minimizes the variance between corresponding (matching) peaks of two different peak lists. The more recently developed Peakmatch algorithm can also match a set of peak lists against a reference peak list and derive offset values using a complete grid search or downhill simplex optimization (Buchner et al. 2013). Both AutoAssign’s registration analysis algorithm and the Peakmatch algorithm work in pairwise mode, i.e. they match a target peak list against a reference peak list, but they are both unable to derive statistics necessary to group peaks into spin systems within a single peak list with more than one peak per spin system (e.g. HN(CO)CACB, NCACX, CANCOCX). While single peak list registration functionality is not required to group peaks into spin systems, it facilitates the development of new grouping algorithms that use a bottom-up approach in grouping peaks into spin systems. In other words, single peak list registration can facilitate the creation of more accurate spin system groups from more reliable smaller variance peak lists first and then extend those spin systems across spectra using pairwise registration statistics derived from pairwise alignment of two different peak lists.

Therefore, we have developed a new registration analysis algorithm that can execute in two modes: (i) a pairwise-registration analysis mode that aligns two different peak lists against each other and calculates necessary dimension-specific peak position statistics; and (ii) a self-registration analysis mode that calculates dimension-specific peak position statistics for a single peak list with multiple peaks per spin system while fixing the alignment to zero. This latter registration analysis mode is accomplished by matching the single peak list against itself while ignoring same-peak matches in order to calculate these dimension-specific peak positional variances. This new registration analysis algorithm provides the necessary statistics to allow both intra- and inter-peak-list peak grouping and to assess the peak positional uncertainty of individual peak lists.

### Application of registration analysis algorithm in grouping algorithm

Since peak positions have multiple sources of variance which are difficult to handle with uniform match tolerances, we also developed a new iterative grouping algorithm that combines our peak list registration analysis algorithm with an adaptation of the density-based spatial clustering of applications with noise (DBSCAN) clustering algorithm normalized by dimension-specific peak position variances. This combined algorithm is capable of grouping peaks from a single peak list into spin systems using different sets of match tolerances derived from our new registration analysis algorithm in an iterative analysis.

### Algorithm for generating simulated peak lists with multiple sources of variance

A related problem is the limited number of assigned experimental peak lists available in the public repositories for the robust evaluation of computational NMR analysis algorithms and methods. As of March, 2017, the Biological Magnetic Resonance Data Bank (BMRB) (Ulrich et al. 2008) contains only a few hundred assigned peak lists from a wide variety of NMR experiments. In order to utilize these assigned peak lists for software tool evaluation, they need to be extracted and converted into appropriate file formats (e.g. Sparky (Goddard and Kneller 2008; Lee et al. 2014), AutoAssign, Xeasy (Bartels et al. 1995), etc.). Also, thorough robustness analysis requires thousands of assigned peak lists for the rigorous testing of algorithms and methods. To provide the necessary datasets, simulated peak lists can be derived from assigned protein resonance assignment entries in the BMRB. However, the simulation of assigned peak lists that provide the same level of difficulty as real experimental peak lists is difficult to generate. Historically, few published methods have been evaluated with simulated peak lists incorporating even a single source of variance. One published evaluation of protein resonance assignment methods even used simulated peak lists with no variance added, representing a very unrealistic test of performance (Wang et al. 2005).

To address these and related NMR-STAR file utilization problems, we have developed the nmrstarlib package (Smelter et al. 2017), a new open source library that can be used to extract experimental peak list data from BMRB entries and convert them into peak lists of appropriate format (e.g. Sparky, AutoAssign, Xeasy). In addition, we developed a peak list simulator that can create peak lists of different types using the entire BMRB, allowing the creation of large numbers of simulated assigned peak lists that includes dimension-specific noise from multiple sources of variance as specified by the user. This new peak list simulator is also part of the nmrstarlib package (Smelter et al. 2017).

## Materials and methods

### Experimental data sets

The combined registration analysis and grouping algorithm was evaluated using 16 different experimental peak lists from 13 different proteins: ten peak lists were derived from solution NMR experiments and six peak lists were derived from solid-state NMR experiments (Table 1). Peak lists usually contain chemical shift values for each dimension that correspond to specific pattern in specific NMR experiment and may contain additional information such as peak intensity, line width, and peak volume.

**Table 1:** Solution and solid-state NMR derived peak lists

Protein	Sequence length	Spectrum type	NMR type	BMRB ID/PDB ID
Bovine pancreatic trypsin inhibitor (BPTI)	58	HN(CO)CACB	Liquid-state	5359/5PTI
Cold shock protein (CspA) (Feng et al. 1998)	70	HN(CO)CACB	Liquid-state	4296/3MEF
Protein yggU from *E.coli* (Target ER14) (Aramini et al.2003a)	108	HN(CO)CACB	Liquid-state	5596/1N91
Fibroblast growth factor (FGF) (Moy et al. 1995)	154	HN(CO)CACB	Liquid-state	4091/1BLD
30S ribosomal protein S28E from *P. horikoshii* (Target JR19) (Aramini2003b)	82	HN(CO)CACB	Liquid-state	5691/1NY4
Non-structural protein 1 (NS1) (Chien et al. 1997)	73	HN(CO)CACB	Liquid-state	4317/1NS1
Ribonuclease pancreatic (RnaseC6572S) (Shimotakahara et al. 1997)	124	HN(CO)CACB	Liquid-state	4032/1SRN
Ribonuclease pancreatic (RnaseWT) (Shimotakahara et al. 1997)	124	HN(CO)CACB	Liquid-state	4031/1SRN
Z domain of staphylococcal protein A (Zheng et al. 2004)	71	HN(CO)CACB	Liquid-state	5656/1H0T
Staphylococcus aureus protein SAV1430 (Target ZR18) (Mereier et al. 2006)	91	HN(CO)CACB	Liquid-state	5844/1PQX
β1 immunoglobulin binding domain of protein G (GB1) (Franks et al. 2005)	56	CANCOCX	Solid-state	15156/2JSV
β1 immunoglobulin binding domain of protein G (GB1) (Franks et al. 2005)	56	NCACX	Solid-state	15156/2JSV
β1 immunoglobulin binding domain of protein G (GB1) (Franks et al. 2005)	56	NCOCX	Solid-state	15156/2JSV
Disulfide bond formation protein B (DsbB) (Tang et al. 2013)	176	NCACX	Solid-state	18493/2LTQ
Cytoskeleton-associated protein-glycine-rich domains (CAP-Gly) (Yan et al. 2013)	89	NCACX	Solid-state	19025/2M02
Cytoskeleton-associated protein-glycine-rich domains (CAP-Gly) (Yan et al. 2013)	89	NCOCX	Solid-state	19025/2M02

### Simulated data sets

Simulated HN(CO)CACB peak lists were generated using our peak list simulation algorithm. For HN(CO)CACB peak lists, every amino acid in the protein sequence not followed by a proline residue should produce two peaks per spin system, except for glycine residues due to missing CB resonances. Using individual entries from BMRB, we generated 6896 “ideal” (0-variance) peak lists using H, N, CA, and CB assigned chemical shifts. Then we filtered out peak lists that had exact duplicate peaks in all three dimensions for every peak, because it would create spin systems with more than two peaks per spin system and mark those spin systems as 100% overlapped due to peak duplicates no matter what variance those peak lists have. Next, we removed peak lists that had missing chemical shift values for CA or CB except for glycine residues because that would decrease the percentage of correctly grouped peaks due to missing data rather than due to increase in dimension-specific variance. Two thousand five hundred forty nine peak lists remained after removing peak lists with duplicate peaks or missing data. Using these remaining peak lists, additional peak lists were simulated for single source of variance in all dimensions, two sources of variance in all dimensions, and two sources of variance in N dimension by adding varying amounts of normally-distributed random noise Eq. 1:

1 $$p\left( x \right)=\frac{1}{{\sqrt {2\pi {\sigma ^2}} }}{e^{ - \frac{{{{\left( {x - \mu } \right)}^2}}}{{2{\sigma ^2}}}}}$$

where $\mu$ is mean, and $\sigma$ is standard deviation. In the case of two sources of variance, 20% of the peaks had noise standard deviation added that is five times larger than 80% of the remaining peaks in each simulated peak list.

### Registration analysis algorithm

Our new registration analysis algorithm is based on a previously developed peak list registration analysis algorithm within the automated protein resonance assignment program AutoAssign (Moseley et al. 2010; Monleón et al. 2002), but which has never been well-described in prior publications. The algorithm has similarities to a point pattern match algorithm (Ranade and Rosenfeld 1980) and a landsat image registration algorithm (Ton and Jain 1989) developed in the 1980s, but solves a more generalized multiple mapping issue than either of those older algorithms. We have made extensive modifications to the algorithm that includes new functionality and significant improvement in the computational efficiency. Our new registration analysis algorithm can perform both pairwise-registration of two different peak lists as well as self-registration of a single peak list that has multiple peaks per spin system. In either algorithmic mode, the registration analysis algorithm operates on two peak lists: an “input” peak list and a “root” or reference peak list. The algorithm calculates the best mapping of peaks from the “input” peak list to peaks in the “root” peak list for their comparable spectral dimensions to derive offsets needed to translate the “input” peak list to the “root” peak list in these comparable dimensions. The algorithm also calculates the standard deviation between mapped pairs of peaks in their comparable dimensions. The self-registration analysis mode of the algorithm treats a single peak list as both the “input” and “root” peak lists and then calculates the best mapping of peaks assuming zero translation offsets and ignoring perfect matches due to self-mapping.

**Fig. 2:**
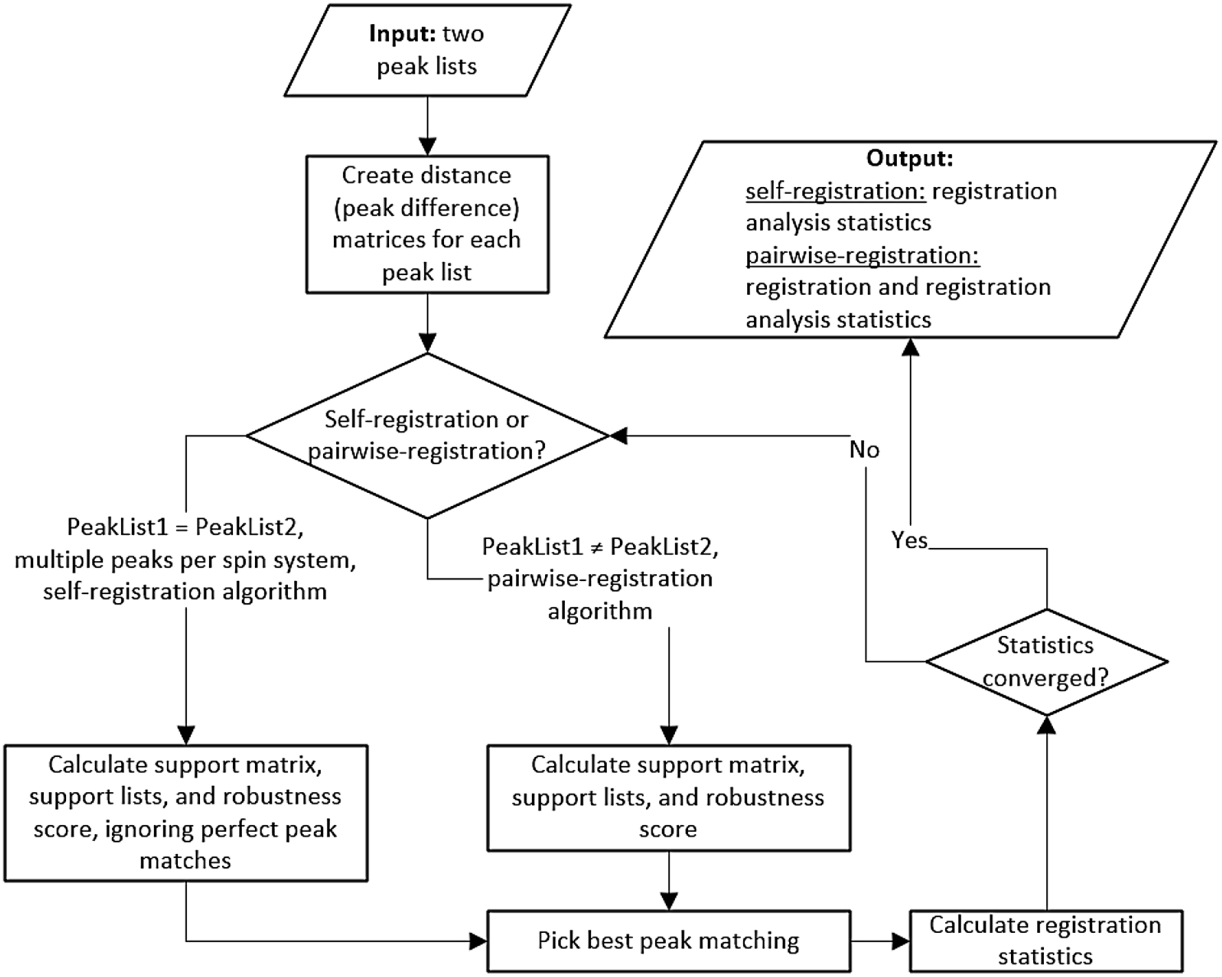
Flow diagram of the peak list registration analysis algorithm

Figure 2 shows the flow diagram of the new registration analysis algorithm for both pairwise- and self-registration execution modes. First, the algorithm parses two peak list files (i.e. the same peak list file twice for self-registration). Then for each peak list, the algorithm constructs a Euclidean distance matrix, i.e. calculates the distance between every pair of peaks within a peak list. If the “input” peak list is identical to the “root” peak list, the self-registration branch of the algorithm executes. If the “input” and “root” peak list are different, the pairwise-registration branch of the algorithm executes. Next, the algorithm creates a support matrix and compares each “input” peak distance matrix row to each “root” peak distance matrix row in order to calculate the set of supporting peak mapping pairs, i.e. the support set (SS). Each cell in the support matrix has a set of support pairs $\left( {m,n} \right) \in ~S{S_{i,j}}$, i.e. pairs of indexes that identify individual coordinates in the support matrix. Using the pair of indexes, a corresponding support set can be identified. Using the support pairs in the support sets, the robustness score for a given support pair (*i, j*) is calculated using a sum of Jaccard similarity coefficients (Jaccard indeces) multiplied by corresponding peak difference matching probabilities as illustrated in Eq. 2:

2 $$robustness(i,j) = \mathop \sum \limits_{{(m,n) \in SS_{{i,j}} }} \frac{{SS_{{i,j}} \mathop \cap \nolimits^{} ~SS_{{m,n}} }}{{SS_{{i,j}} \mathop \cup \nolimits^{} ~SS_{{m,n}} }} \cdot p_{{\chi _{{df}}^{2} \left( {i,j,m,n} \right)}}$$

where $i,j$ are the row and column coordinates of the support matrix, $m,n$ are the row and column coordinates of the support matrix whose pair $\left( {m,n} \right)$ is an element of $S{S_{i,j}}$, and ${p_{{\chi ^2},~df}}$ is the Chi square probability calculated for corresponding peak differences in the input and root peak lists for specified degrees of freedom $df$, i.e. as defined by Eq. 3:

3 $$\chi _{{df}}^{2}\left( {i,j,m,n} \right)=\mathop \sum \limits_{l=0}^{df} {\left( {\frac{{\left( {input~peak~lis{t_i}\left[ l \right] - input~peak~lis{t_m}\left[ l \right]} \right) - \left( {root~peak~lis{t_j}\left[ l \right] - root~peak~lis{t_n}\left[ l \right]} \right)}}{{std\left[ l \right] \cdot 2}}} \right)^2}$$

where $l$ specifies the index of the comparable dimension of a peak in both the input and root peak lists and their corresponding standard deviation $std$. A supporting peak mapping pair is determined by a match tolerance defined in terms of standard deviation units. The default is four standard deviation units. The self-registration execution mode excludes identical peak mappings from this comparison. Using the support list, a robustness score is calculated for each comparison. The robustness score indicates how many peaks in the “input” peak list are mapped to corresponding peaks in “root” peak list in a concordant manner (i.e. below match tolerances) with a single mapping peak-pair representing the center of the concordance. The higher the robustness score, the larger the concordance. Next, the algorithm uses the support list of the peak mapping pair with the best robustness score to calculate the registration offsets and statistics, which is used to derive new match tolerances. The algorithm iterates until the statistics of registration converge, i.e. until per dimension standard deviations stop changing.

One detail to note in Eq. 3 is the use of $std[l] \cdot 2$ in calculating the chi-squared statistic. Based on linear error analysis and independent variable propagation rules, one would expect $std[l] \cdot \sqrt 2$ to be the correct estimate of the standard deviation to use in this equation. However, in this iterative registration approach, $std[l] \cdot 2$ provides superior performance (See Supplemental Tables S1, S2). We believe that the use of 2 instead of $\sqrt 2$ accounts for non-independent error propagation in the given difference of differences analysis.

### Grouping algorithm

Our single peak list spin system grouping algorithm is based on the widely-used density-based clustering algorithm DBSCAN (Ester et al. 1996), which can detect clusters of varying size and shape. The original DBSCAN algorithm requires two global parameters: radius ε, which defines ε-neighborhood of a point and minimum number of points, µ that can form a cluster. The DBSCAN algorithm uses a region query similarity function to initialize clusters where it calculates the Euclidean distance between core point and every other point in the data and function that expands cluster by examining neighborhoods of points in initialized cluster in order to discover cluster points (Ester et al. 1996).

In our case, each peak represents a point in a peak list data and in order to group peaks into clusters (spin systems) without overlap or split, we would have to know the radius ε for each of the clusters in advance. For peak list data, it is not easy to know those parameters in advance and requires domain expert to identify tolerances needed for grouping peaks into spin systems (clusters). This is further complicated by the presence of multiple sources of variance affecting subsets of peaks within a single peak list, i.e. some peaks will require larger tolerances for grouping them into spin systems than others. Therefore, uniform tolerances cannot be used to discover optimal peak grouping.

For our grouping algorithm, we replaced the region query function that uses neighborhood radius ε and the Euclidean distance similarity function with versions that use a hi-squared-based distance cutoff and variance-normalized distance (hi-squared statistic) to decide if a peak can be included into a spin system cluster or not. These statistics-inspired changes create a variance-informed version of the DBSCAN algorithm. Equation 4 describes the criteria for inclusion or exclusion of peaks from initialized spin system cluster:

4 $$\left\{ {\begin{array}{*{20}c} {\sqrt {\mathop \sum \limits_{{k = 0}}^{{df}} \left( {\frac{{peak_{i} \left[ k \right] - peak_{j} \left[ k \right]}}{{std\left[ k \right]}}} \right)^{2} } \le \sqrt {F^{{ - 1}} \left( {p,~df} \right)} ~} \\ {\sqrt {\mathop \sum \limits_{{k = 0}}^{{df}} \left( {\frac{{peak_{i} \left[ k \right] - peak_{j} \left[ k \right]}}{{std\left[ k \right]}}} \right)^{2} }> \sqrt {F^{{ - 1}} (p,~df)} } \\ \end{array} } \right.~$$

where $pea{k_i}$ and $pea{k_j}$ is every pair of peaks within a single peak list,$df$—number of degrees of freedom that correspond to the number of comparable dimensions, $k$—specifies index of comparable dimension within a peak and its corresponding standard deviation $std$ obtained from the registration analysis algorithm, ${F^{ - 1}}(p,~df)$—chi-squared inverse cumulative distribution function for a given $p$-value and degrees of freedom. If the normalized distance between peaks is less or equal than the inverse survival function for a given $p$-value and corresponding degrees of freedom, the peak belongs to the spin system cluster, otherwise the peak is excluded from the spin system cluster. The variances used to calculate the normalized distance are supplied by our self-registration analysis algorithm. The use of a chi-squared statistic allows the cutoff parameter to be provided in terms of a chi-squared-based probability. The default for the algorithm is a $p$-value = 0.0001.

**Fig. 3:**
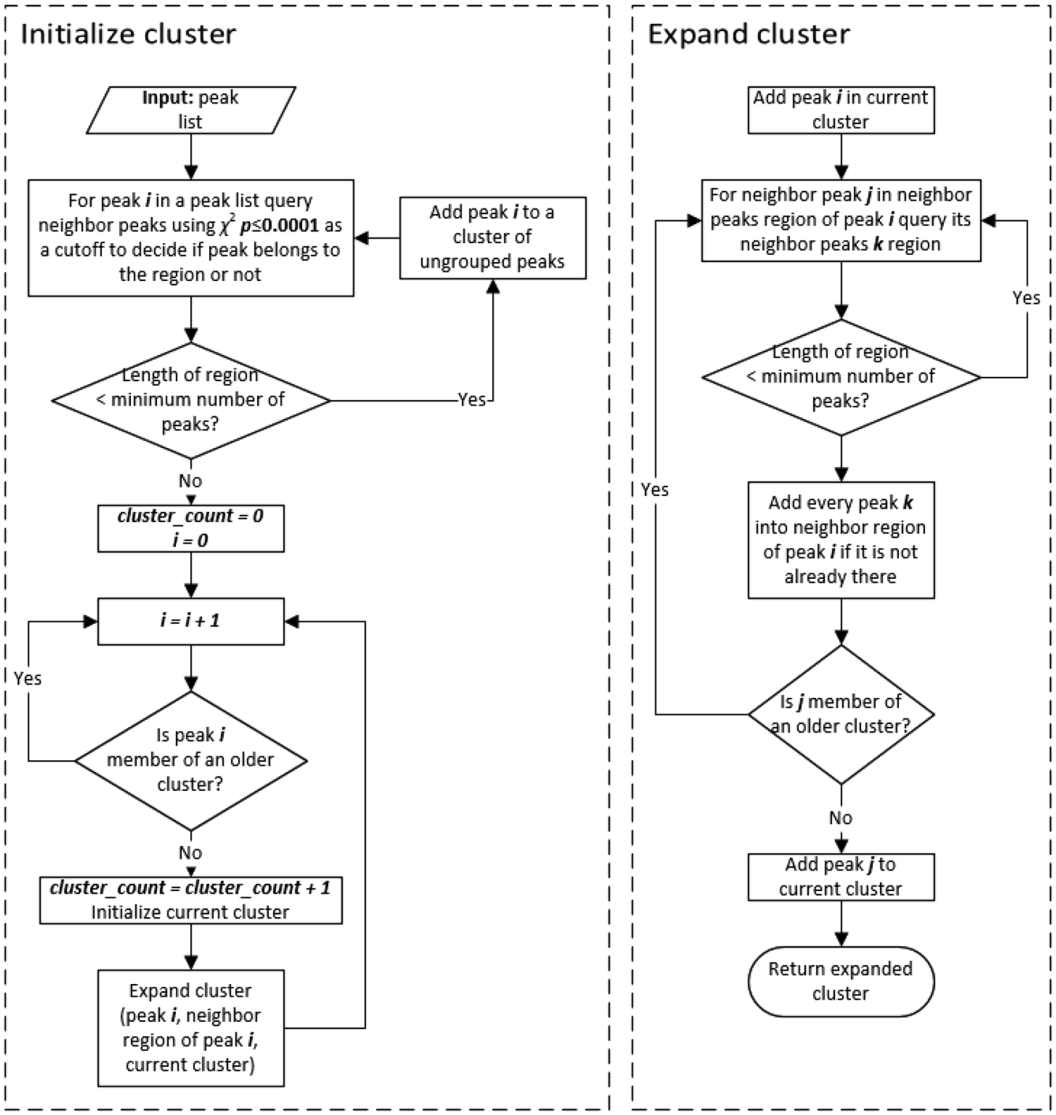
Flow diagram of the grouping algorithm

Figure 3 shows the flow diagram of the peak grouping algorithm that groups peaks within a single peak list into spin systems. The grouping algorithm consists of two main functions—one that initializes the clusters and the other that expands clusters by examining the neighborhood of an initialized cluster in a similar fashion to DBSCAN (Ester et al. 1996).

### Combined registration analysis and grouping algorithm

**Fig. 4:**
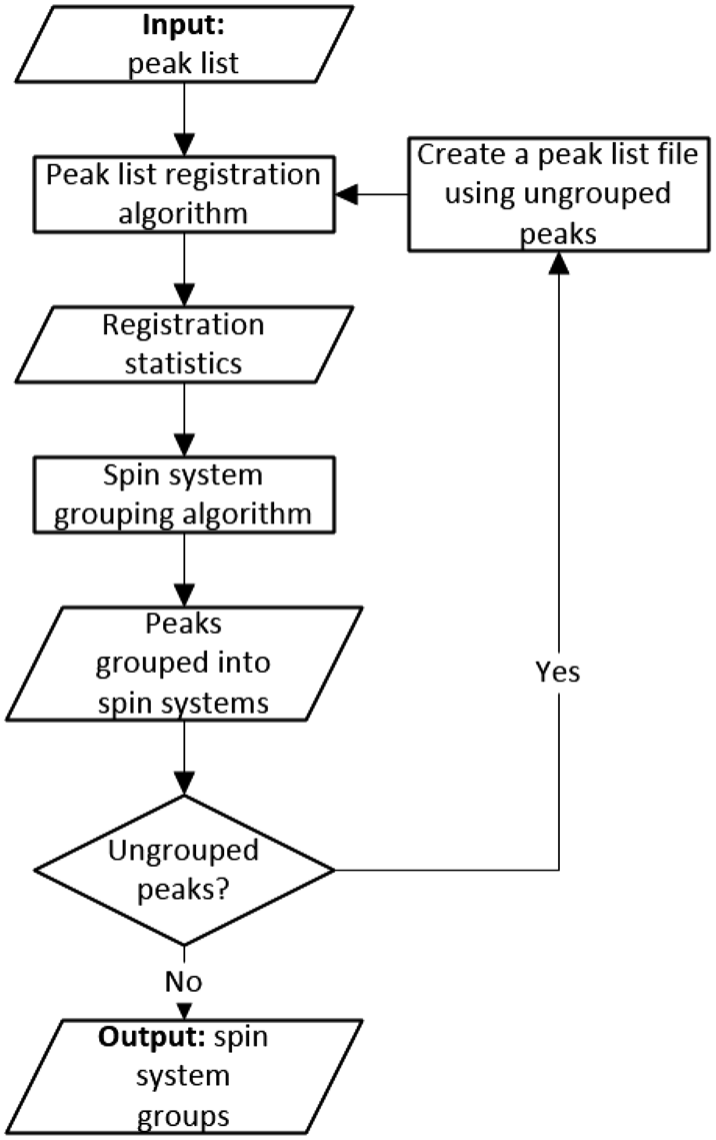
Flow diagram overview of the entire registration analysis and grouping process

In order to address the presence of multiple sources of peak positional variance, we developed an iterative algorithm that combines both the self-registration analysis algorithm and grouping algorithm to derive spin system clusters using multiple variance-based match tolerances calculated with the help of the registration analysis algorithm. Figure 4 shows the flow diagram of combined algorithm. First, the combined algorithm reads a single peak list in and runs the self-registration analysis algorithm to identify initial variance values for each comparable dimension. Next, the grouping algorithm uses per dimension variance values to group peaks into spin system clusters. Then, the combined algorithm checks if there are unclustered peaks left. From the unclustered peaks, the algorithm creates a new peak list file and attempts to register it against itself again to determine new larger variances that can be used to group peaks into spin system clusters. Conceptually, the iterative algorithm poses the problem in terms of a linear mixture of multiple normal-like distributions that create groups of peaks that have variable positional density.

### Peak list simulation algorithm

**Fig. 5:**
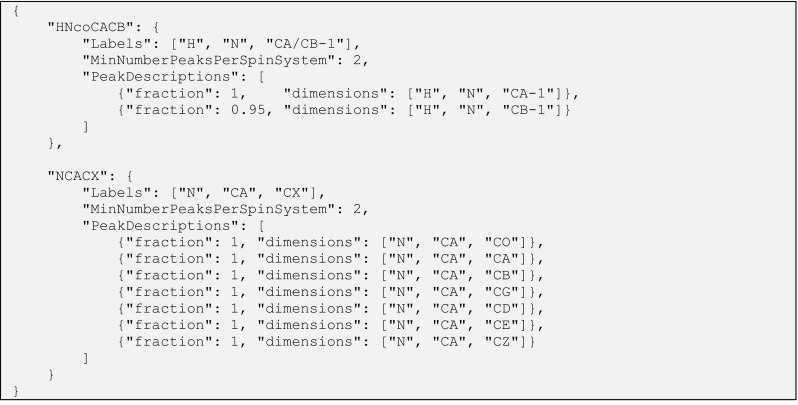
Spectrum description configuration file of peak list simulation algorithm

To create additional data sets for robustness analysis, we developed an algorithm that can simulate peak lists using assigned chemical shift values deposited in BMRB entries. We implemented this algorithm as a peak list simulator submodule within the previously developed nmrstarlib Python package (Smelter et al. 2017), which facilitates the reading and writing of NMR-STAR formatted files, especially entry files maintained by BMRB. This algorithm uses the nmrstarlib functionality to access assigned chemical shift values for H, C and N resonances for each residue in a protein chain and then saves them as a peak list file in different formats (e.g. Sparky, AutoAssign, JSON). Moreover, the algorithm provides the ability to add varying amounts of noise to each dimension of the peak list in order to create more realistic data sets. The peak list simulator uses a very generic spectrum definition based on different resonance classes (e.g. CA, CB, N, etc.) and their relative positions (−1, 0, +1, etc.): therefore, different through-bond experiments can be described for both solution and solid-state NMR spectra very easily. The local contact peaks for through-space experiments can be simulated as well using the relative position descriptions (0, +1, +2, +3, +4). Figure 5 shows an example of a spectrum description configuration file in javascript object notation (JSON) format. This design allows a user to easily add new experiment descriptions using this configuration file without hardcoding peak list creation logic into source code.

### Programming details, command-line interfaces, and availability

All developed algorithms can be used as stand-alone tools and have simple command-line interfaces. The registration analysis algorithm was implemented using the C++ programming language using the C++11 standard. The grouping and peak list simulation algorithms were implemented using the latest version of the Python programming language (currently 3.6.0). Supplementary Fig. S1–S3 show command-line interfaces for registration, grouping, and peak list simulation algorithms respectively.

The software package that implements the single peak list registration analysis and grouping algorithms is available under figshare repository (10.6084/m9.figshare.4814605), documentation with examples (10.6084/m9.figshare.4816441), solution-state and solid-state NMR experimental peak lists (10.6084/m9.figshare.4815163), simulated peak lists (10.6084/m9.figshare.5260660), results of the registration analysis and grouping algorithms and visualization of results (10.6084/m9.figshare.4815160).

The nmrstarlib package is available at http://software.cesb.uky.edu, at GitHub (https://github.com/MoseleyBioinformaticsLab/nmrstarlib) and at PyPI (https://pypi.python.org/pypi/nmrstarlib) under the MIT license. Project documentation is available online at ReadTheDocs (http://nmrstarlib.readthedocs.io/).

## Results and discussion

### Performance on experimental data sets

First, we evaluated the performance of our combined registration analysis and grouping algorithm on manually assigned peak lists derived from solution and solid-state NMR experiments. Table 2 shows the summary of results for peak lists derived from solution NMR HN(CO)CACB type experiments (Grzesiek and Bax 1992). The expected number of peaks for the HN(CO)CACB peak list can be estimated from a protein sequence, i.e. for every spin system in a protein there should be at least two peaks except for glycine (due to missing CB resonance) and proline (due to missing amide H resonance) residues ([number of amino acids in sequence − number of prolines − number of glycines] × 2 + number of glycines − 1). Similarly, the expected number of spin systems (clusters) for the HN(CO)CACB peak list can be estimated from a known sequence (number of amino acids in sequence − 1 − number of GLY residues − number of PRO residues). The number of observed peaks is usually larger than the number of expected peaks for a given protein sequence due to NMR artefacts. The number of ungrouped peaks shows how many peaks were left ungrouped after iterative registration analysis and grouping procedure. This number is proportional to number of glycine residues (because of missing corresponding peak for the CB resonance) in the protein sequence, and the number of artefact peaks that appear in the spectrum. The numbers of missing, overlapped, and split spin systems were inferred directly from the assigned peak lists. For example, a split in spin systems occurs when two peaks that should form their own spin system cluster end up being added into other neighbor spin system clusters. Results of our iterative grouping algorithm summarized in Table 2 show that it is capable of grouping peaks into spin system clusters that correspond to real spin systems in a protein sequence. When we limited our grouping algorithm to a single registration-grouping iteration, the number of identified clusters decreased dramatically (See Table 2 value in parenthesis) ranging from 13% less recovered clusters for 30S ribosomal protein (BMRBID 5691) to 57% less recovered clusters for ribonuclease pancreatic (BMRBID 4032).

**Table 2:** Spin system grouping results for solution NMR derived peak lists using combined registration analysis and grouping algorithm

Protein / Peak list	Expected peaks	Observed peaks	Ungrouped peaks	Expected spin systems	Identified spin systems^a^	Missing spin systems	Overlapped spin systems	Split spin systems
BPTI/HN(CO)CACB	101	134	17	47	54 (30)	0	0	2
CSP/HN(CO)CACB	125	145	39	57	53 (32)	12	0	0
ER14/HN(CO)CACB	194	181	7	93	87 (57)	8	2	0
FGF/HN(CO)CACB	273	303	24	128	139 (112)	13	2	1
JR19/HN(CO)CACB	151	141	7	71	67 (58)	4	0	0
NS1/HN(CO)CACB	137	203	36	66	81 (43)	26	8	2
RnaseC6572S/HN(CO)CACB	235	282	16	116	130 (56)	18	4	2
RnaseWT/HN(CO)CACB	235	403	19	116	181 (122)	9	2	1
ZDOM/HN(CO)CACB	134	153	29	67	55 (40)	15	3	5
ZR18/HN(CO)CACB	172	163	3	85	80 (52)	5	0	0

^a^Value in parenthesis shows how many spin systems were identified if only uniform tolerances were used and single iteration of grouping algorithm was performed

Table 3 contains similar summary results for solid-state NMR derived peak lists. CANCOCX (Franks et al. 2007), NCACX (Pauli et al. 2001), and NCOCX (Pauli et al. 2001) peak lists for the GB1 protein were nearly complete and therefore showed low number of overlapped and split spin systems. Peak lists for DsbB and Cap-Gly proteins had a large number of missing and artefact peaks, therefore we observed a higher number of overlapped and split spin systems. The quality of peak list registration analysis and therefore spin system grouping is highly correlated with the quality of peak lists. Also, the larger the number of missing and artefact peaks in the peak lists, the larger the number of overlap in spin systems that were generally observed. Similar to solution NMR derived peak lists, we limited the algorithm to a single registration-grouping iteration. However, we observed that solid-state NMR derived peak lists were more consistent and did not have as much dimension-specific variance in comparison to solution NMR derived peak lists (See Table 3 value in parenthesis). This may seem surprising, given the typical lower spectral quality of solid-state NMR spectra in comparison to solution NMR spectra in terms of sensitivity and peak widths. However, when good quality solid-state NMR spectra are obtainable, the greater spread of peaks across ^15^N and ^13^C dimensions used for grouping provides advantages over the more crowded amide ^1^H and ^15^N dimensions used for grouping in solution NMR spectra.

**Table 3:** Spin system grouping results for solid-state NMR derived peak lists using combined registration analysis and grouping algorithm

Protein/Peak list	Expected peaks^a^	Observed peaks	Ungrouped peaks	Expected spin systems	Identified spin systems^b^	Missing spin systems	Overlapped spin systems	Split spin systems
GB1/CANCOCX	268	240	70	55	56 (56)	1	6	28
GB1/NCACX	268	463	62	55	65 (65)	0	0	19
GB1/NCOCX	268	474	16	55	82 (67)	0	4	10
DsbB/NCACX	940	215	43	175	47 (47)	126	14	1
CapGly/NCACX	410	515	16	88	50 (50)	33	25	0
CapGly/NCOCX	410	218	25	88	47 (47)	38	32	5

^a^Number of expected peaks estimated based on magnetization transfer pattern and amino acid sequence. Alternative magnetization transfer pathways increase the number of peaks present

^b^Value in parenthesis shows how many spin systems were identified if only uniform tolerances were used and single iteration of grouping algorithm was performed

**Fig. 6:**
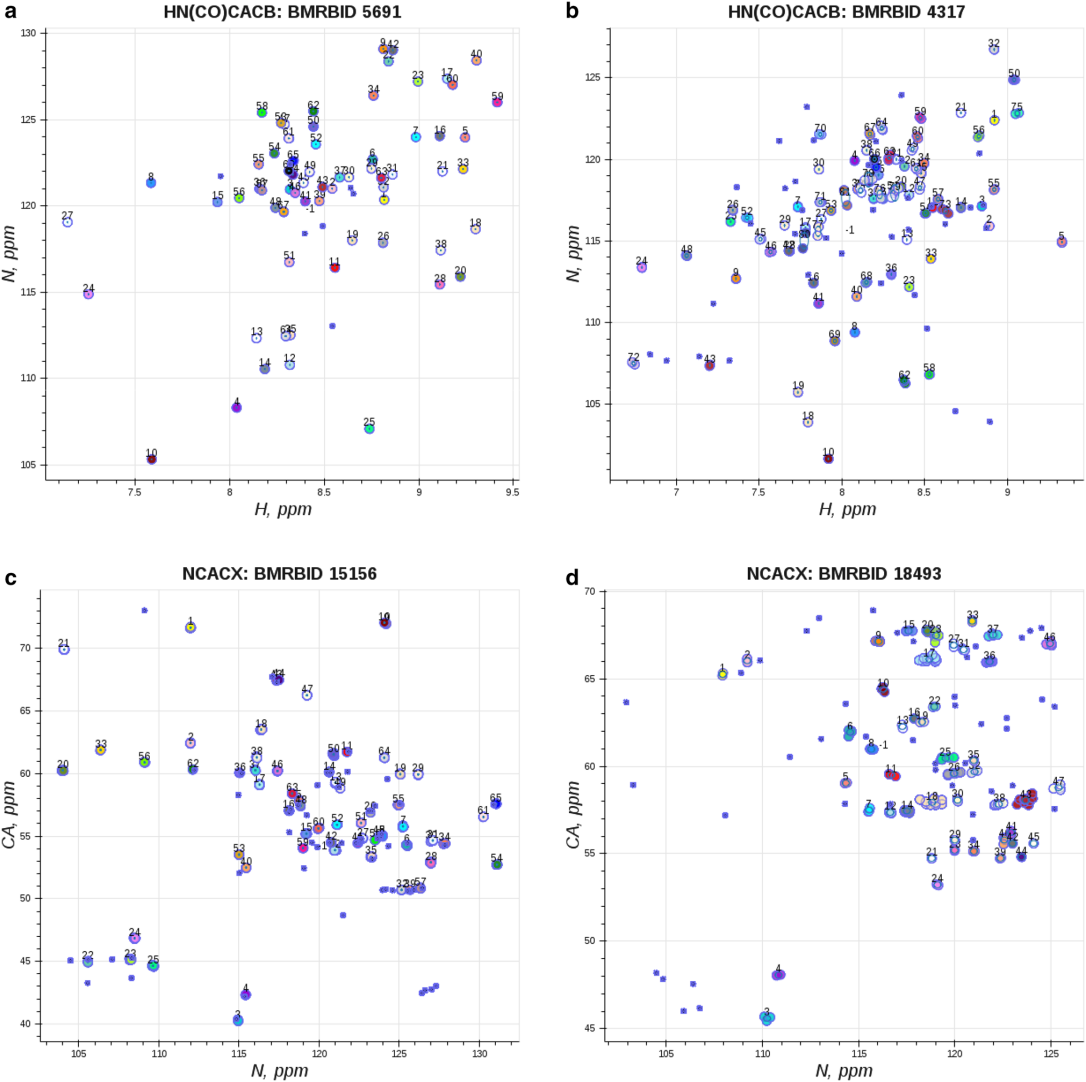
Visualization of spin system grouping results where *colored points* correspond peak centers grouped into spin systems, peak centers of the *same color* belong to the same spin system (spin systems are numbered sequentially), unnumbered *blue points* correspond to either spurious unassigned peaks or in case of HN(CO)CACB peak lists peaks corresponding to glycine residues (due to missing CB resonance): **a** example of best spin system clustering for 30S ribosomal protein S28E from *P. horikoshii* (HN(CO)CACB peak list); **b** example of worst spin system clustering non-structural protein 1 (HN(CO)CACB peak list); **c** example of best spin system clustering for GB1 protein (NCACX peak list); **d** example of worst spin system clustering for DsbB protein (NCACX peak list)

Best and worst spin system grouping results are visualized in Fig. 6. Panel a shows the best grouping result for solution NMR derived peak lists for 30S ribosomal protein S28E from *P. horikoshii* where clean non-overlapped spin system clusters are formed (numbered points of different color), and small number of artefact peaks are present (smaller unnumbered points); panel b shows the worst result for solution NMR derived peak lists for non-structural protein 1, which has more overlap (spin system clusters #73, #77, #79, and #80) and significantly larger number of artefact peaks (smaller unnumbered points); panel c shows the best grouping result among the solid-state NMR peak lists for GB1 protein where no overlap is present within spin system groups; and panel d shows the worst result among solid-state NMR peak lists for DsbB protein, with more artefact peaks observed in comparison to solution NMR peak lists and significantly higher overlap due to the lower quality of the peak list (spin system clusters #9, #13, #17, #18, #25, #29).

### Performance on simulated data sets

**Fig. 7:**
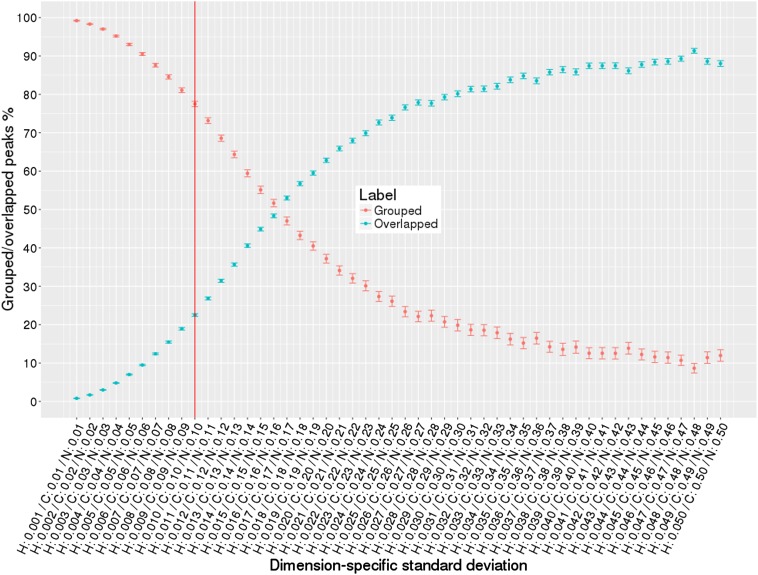
Single source of variance in all dimensions: percentage of grouped (non-overlapped) and overlapped peaks with increase in standard deviation values of peak dimensions. The *dots* correspond to the percentage of the grouped/overlapped peaks, *whiskers* are calculated standard error of the mean

**Fig. 8:**
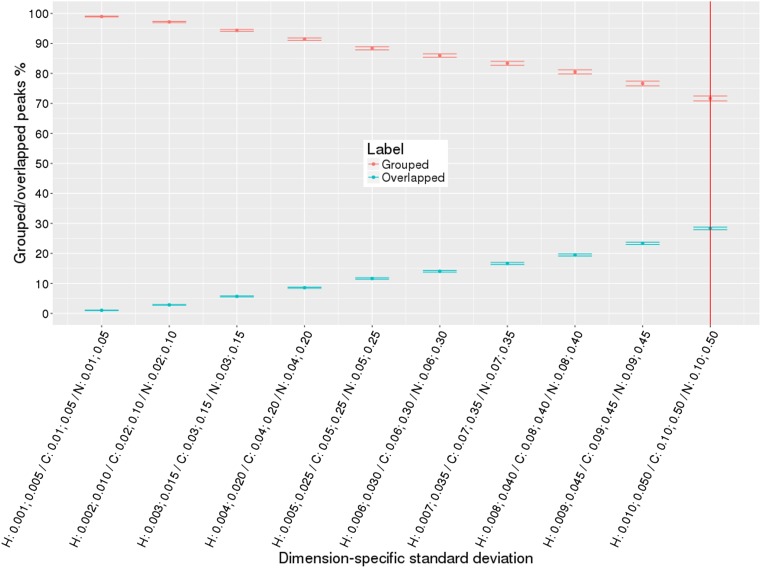
Two sources of variance in all dimensions: percentage of grouped (non-overlapped) and overlapped peaks with increase in standard deviation values of peak dimensions, 20% of peaks have five times larger variance than the remaining 80% of peaks in all dimensions. The *dots* correspond to the percentage of the grouped/overlapped peaks, *whiskers* are calculated standard error of the mean

**Fig. 9:**
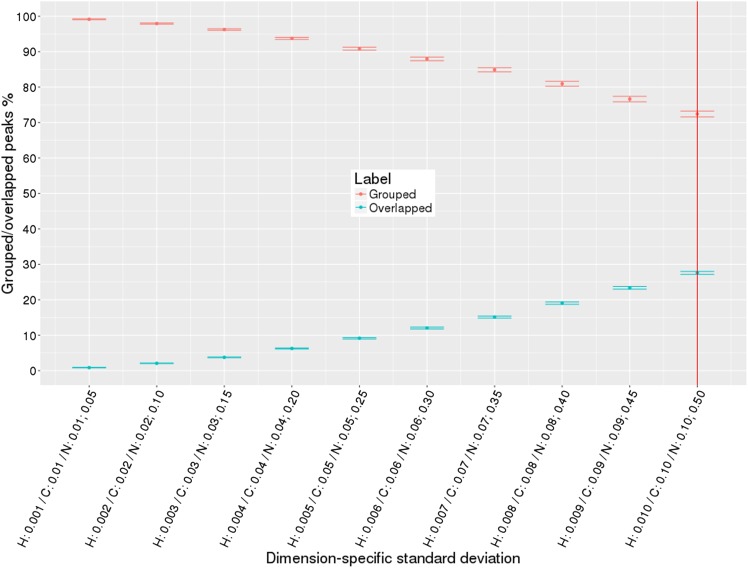
Two sources of variance in one dimension: percentage of grouped (non-overlapped) and overlapped peaks with increase in standard deviation values of peak dimensions, 20% of peaks have five times larger variance than the remaining 80% of peaks in N dimension. The *dots* correspond to the percentage of the grouped/overlapped peaks, whiskers are calculated standard error of the mean

To evaluate robustness of our algorithms, we generated large numbers of simulated HN(CO)CACB peak lists (see Table 4). To create peak lists that better reflect what is observed in experimental peak lists, we introduced varying amounts of noise based on random normal distributions for several conditions: (i) single source of variance in all dimensions; (ii) two sources of variance in all dimensions; (iii) two sources of variance in one dimension. Figure 7 (See Supplementary Figs. S4, S5) demonstrates results for single source of variance condition, where we simulated peak lists with increasing random noise from 0.001 to 0.050 for ^1^H dimension and from 0.01 to 0.50 for ^13^C and ^15^N dimensions. The percentage of accurately grouped peaks versus percentage of overlapped peaks are plotted as a function of dimension-specific standard deviations. The red vertical line separates high quality versus low quality peak lists with larger peak positional variance and overlap. Normally, good quality peak lists have ^1^H, ^13^C, and ^15^N chemical shift standard deviations on the left side of the red line. It is clear from the diagram that for the smallest variance in peak positions, our algorithm groups 99% of peaks into correct non-overlapped spin systems across all simulated peak lists. As variance in peak positions increases percentage of overlapped peaks increases. At larger dimension-specific variance condition (0.01 for ^1^H dimension and 0.1 for ^13^C and ^15^N dimensions), it is still capable of grouping 77% of peaks into clean non-overlapped spin systems. Figure 8 (See Supplementary Figs. S6, S7) shows similar results but for two sources of variance in all dimensions, i.e. 80% of peaks had random normal noise added from 0.001 to 0.01 for ^1^H dimension and from 0.01 to 0.1 for ^13^C and ^15^N dimensions, the remaining 20% of peaks had random normal noise five times higher (from 0.005 to 0.05 for ^1^H dimension and from 0.05 to 0.5 for ^13^C and ^15^N dimensions). Figure 9 (See Supplementary Figs. S8, S9) shows results for two sources of variance for only ^15^N dimension, ^1^H and ^13^C had single source of variance. Results on Figs. 8, 9 demonstrate that our iterative grouping algorithm can handle peak lists with multiple sources of variance in single or all dimensions and can group 99% of peaks for the smallest variance values in peak dimensions and 71% of peaks at the 0.01 ^1^H chemical shift standard deviation level.

**Table 4:** Simulated HN(CO)CACB peak lists

Number of variance sources	Minimum standard deviation values	Maximum standard deviation values	Total number of simulated peak lists
Single source of variance in all dimensions	H: 0.001	H: 0.050	127,450
	C: 0.01	C: 0.50	
	N: 0.01	N: 0.50	
Two sources of variance in all dimensions	H: 0.001, 0.005	H: 0.010, 0.050	25,490
	C: 0.01, 0.05	C: 0.10, 0.50	
	N: 0.01, 0.05	N: 0.10, 0.50	
Two sources of variance in N dimension, single source of variance in C and H dimensions	H: 0.001	H: 0.010	25,490
	C: 0.01	C: 0.10	
	N: 0.01, 0.05	N: 0.10, 0.50	

### Comparison to hierarchical DBSCAN

In order to test if other clustering algorithms can be used to group peaks within single peak list into spin system clusters, we used a recently developed variation of DBSCAN called hierarchical DBSCAN (HDBSCAN) (Campello et al. 2013; McInnes et al. 2017). We chose this clustering algorithm, because it has a few advantages: it does not require specification of the expected number of clusters upfront as opposed to k-means clustering algorithm and it does not require specification of the ε-neighborhood parameter as opposed to regular DBSCAN clustering algorithm. This hierarchical version performs DBSCAN using varying values of radius ε and integrates all results to find the best clustering solution. The HDBSCAN algorithm is designed to overcome one of the hardest problems in clustering such as the detection of variable density clusters. Table 5 shows results of HDBSCAN for solution NMR peak lists. The number of overlapped spin systems was significantly higher in comparison to our implementation of combined registration analysis and grouping algorithm when applied to the peak list data. Also, for solid-state NMR derived peak lists HDBSCAN performed slightly worse than our algorithm (see Table 6). Our grouping algorithm implementation is slower than HDBSCAN due to the complexity of the registration analysis algorithm step, but it produces more accurate and more consistent results for both solution and solid-state NMR derived experimental peak lists as well as for simulated peak lists.

**Table 5:** Spin system grouping results for solution NMR derived peak lists using HDBSCAN algorithm

Protein / Peak list	Expected peaks	Observed peaks	Ungrouped peaks	Expected spin systems	Identified spin systems	Missing spin systems	Overlapped spin systems	Split spin systems
BPTI/HN(CO)CACB	101	134	15	47	24	0	31	0
CSP/HN(CO)CACB	125	145	37	57	21	12	35	1
ER14/HN(CO)CACB	194	181	33	93	26	8	77	1
FGF/HN(CO)CACB	273	303	43	128	53	13	108	3
JR19/HN(CO)CACB	151	141	18	71	23	4	66	3
NS1/HN(CO)CACB	137	203	49	66	31	26	43	8
RnaseC6572S/HN(CO)CACB	235	282	38	116	45	18	90	4
RnaseWT/HN(CO)CACB	235	403	68	116	68	9	75	9
ZDOM/HN(CO)CACB	134	153	22	67	25	15	49	5
ZR18/HN(CO)CACB	172	163	42	85	22	5	59	0

**Table 6:** Spin system grouping results for solid-state NMR derived peak lists using HDBSCAN algorithm

Protein/Peak list	Expected peaks^a^	Observed peaks	Ungrouped peaks	Expected spin systems	Identified spin systems	Missing spin systems	Overlapped spin systems	Split spin systems
GB1/CANCOCX	268	240	16	55	51	1	29	9
GB1/NCACX	268	463	14	55	63	0	2	1
GB1/NCOCX	268	474	14	55	67	0	4	7
DsbB/NCACX	940	215	27	175	37	126	31	3
CapGly/NCACX	410	515	36	88	70	33	21	17
CapGly/NCOCX	410	218	20	88	42	38	46	7

^a^Number of expected peaks estimated based on magnetization transfer pattern and amino acid sequence. Alternative magnetization transfer pathways increase the number of peaks present

## Conclusions

Firstly, we have developed a complimentary pair of registration analysis and grouping algorithms that work on a single peak list in order to derive peaks that belong to the same spin system. The new peak list registration analysis algorithm is capable of executing in two modes: self-registration analysis and pairwise-registration analysis. The self-registration analysis mode allows the derivation of registration statistics for a single unassigned peak list that has multiple peaks per spin system.The pairwise-registration analysis mode allows alignment of two different unassigned peak lists in order to calculate registration statistics. Next, the new bottom-up iterative grouping algorithm that can group peaks into spin systems within a single peak list and can handle multiple sources of variance that are present within experimental data sets. Each of the iterations in our grouping algorithm is based on a density-based clustering algorithm with a variance-normalized distance function for calculating which peaks are clustered together, using dimension-specific variances that are derived from the self-registration analysis algorithm. Utilization of the single peak list registration analysis algorithm will facilitate the development of more sophisticated and automated spin system grouping algorithms that produce more accurate spin systems for downstream data analyses.

Secondly, we have developed automated tools that allow the creation of simulated peak lists with a range of positional variances using assigned chemical shifts in BMRB entries. We used these tools to create a very large simulated dataset from the entire BMRB to rigorously test the performance and robustness of our algorithms. These tests showed that our algorithms can detect multiple sources of variance introduced into simulated data sets and reliably group peaks into spin systems for peak lists that are far from ideal.

## Electronic supplementary material

Below is the link to the electronic supplementary material.

Supplementary material 1 (DOCX 244 KB)

Supplementary material 2 (PDF 174 KB)
