# Supplementary material for: Detecting and accounting for multiple sources of positional variance in peak list registration analysis and spin system grouping
Source: J Biomol NMR. 2017 Aug 16;68(4):281–96. doi: 10.1007/s10858-017-0126-5 (PMC5587626; doi:10.1007/s10858-017-0126-5)
Supplement: Supplementary file 1 — Supplementary material 1 (DOCX 244 KB) [file 10858_2017_126_MOESM1_ESM.docx]

| crs (calculate registration statistics) command-line interface  Calculates the registration statistics that will make input_peaklist match root_peaklist (reference peak list).  Usage:  crs (<input_peaklist> <root_peaklist>) [options]  input_peaklist The peak list you wish to register and filter.  root_peaklist The reference peak list.  Options:  --verbose Print more information.  --noi Run in self-registration mode.  --nobounds Do not perform bounds checking.  --dim <i1> <i2> <...> : <r1> <r2> <...> Description of matching dimensions in input and root peak lists.  --tolerance <num_units> Number of stds to use as the match tolerance [default: 4].  --H <init_std> Set starting std to try for H dimensions [default: 0.0075].  --C <init_std> Set starting std to try for C dimensions [default: 0.075].  --N <init_std> Set starting std to try for N dimensions [default: 0.075].  --i <max> Maximum number of iteration to perform [default: 20].  --save <json_filename> Save results of the registration algorithm into JSON file. |
| --- |
| **Figure S1.** *Command-line interface of the registration analysis algorithm.* |

| ssc (Spin System Creator) command-line interface  Usage:  ssc -h \| --help  ssc --version  ssc group (--plpath=<path>) (--plformat=<format>) (--stype=<type>) (--dims=<labels>) (--rdims=<labels>)  [--result=<path>] [--crs=<path>]  ssc visualize <grouping_result> <x_idx> <y_idx> <x_label> <y_label> <plot_title>  Options:  -h, --help Show this screen.  --version Show version.  --plpath=<path> Path to peak list.  --plformat=<format> Peak list format.  --stype=<type> Spectrum type.  --dims=<labels> Comma-separated dimension labels.  --rdims=<labels> Comma-separated root dimension labels.  --crs=<path> Registration algorithm executable path [default: ssc/bin/CRS_EXE]  --result=<path> Path to directory where results will be saved. |
| --- |
| **Figure S2.** *Command-line interface of the Spin System Creator (the combined registration analysis and grouping algorithm).* |

| nmrstarlib command-line interface  Usage:  nmrstarlib -h \| --help  nmrstarlib --version  nmrstarlib convert (<from_path> <to_path>) [--from_format=<format>] [--to_format=<format>]  [--bmrb_url=<url>] [--nmrstar_version=<version>] [--verbose]  nmrstarlib csview <starfile_path> [--amino_acids=<aa>] [--atoms=<at>] [--csview_outfile=<path>]  [--csview_format=<format>] [--bmrb_url=<url>] [--nmrstar_version=<version>] [--verbose]  nmrstarlib plsimulate (<from_path> <to_path> <spectrum>) [--from_format=<format>]  [--to_format=<format>] [--plsplit=<%>] [--H_std=<std>] [--C_std=<std>]  [--N_std=<std>] [--H_mean=<mean>] [--C_mean=<mean>] [--N_mean=<mean>]  [--bmrb_url=<url>] [--nmrstar_version=<version>]  [--spectrum_descriptions=<path>] [--verbose]  Options:  -h, --help Show this screen.  --version Show version.  --verbose Print what files are processing.  --from_format=<format> Input file format, available formats: nmrstar, json [default: nmrstar].  --to_format=<format> Output file format, available formats: nmrstar, json [default: json].  --nmrstar_version=<version> Version of NMR-STAR format to use, available: 2, 3 [default: 3].  --bmrb_url=<url> URL to BMRB REST interface  [default: http://rest.bmrb.wisc.edu/bmrb/NMR-STAR3/].  --amino_acids=<aa> Comma-separated amino acid three-letter codes.  --atoms=<at> Comma-separated BMRB atom codes.  --csview_outfile=<path> Where to save chemical shifts table.  --csview_format=<format> Format to which save chamical shift table [default: svg].  --plsplit=<%> How to split peak list into chunks by percent [default: 100].  --spectrum_descriptions=<path> Path to custom spectrum descriptions file.  --distribution=<func> Statistical distribution function [default: normal].  --H=<value> Statistical distribution parameter(s) for H dimension [default: 0].  --C=<value> Statistical distribution parameter(s) for C dimension [default: 0].  --N=<value> Statistical distribution parameter(s) for N dimension [default: 0]. |
| --- |
| **Figure S3.** *Command-line interface of the peak list simulation algorithm.* |


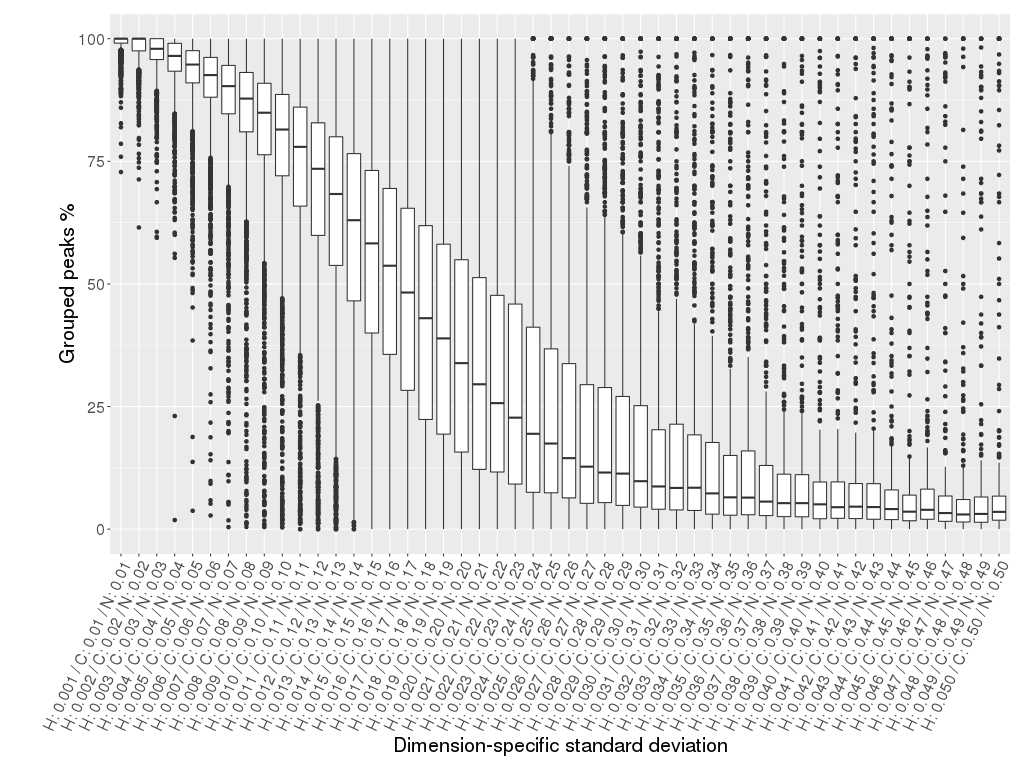


**Figure S4**. Single source of variance in all dimensions: percentage of grouped (non-overlapped) peaks with increase in standard deviation values of peak dimensions.


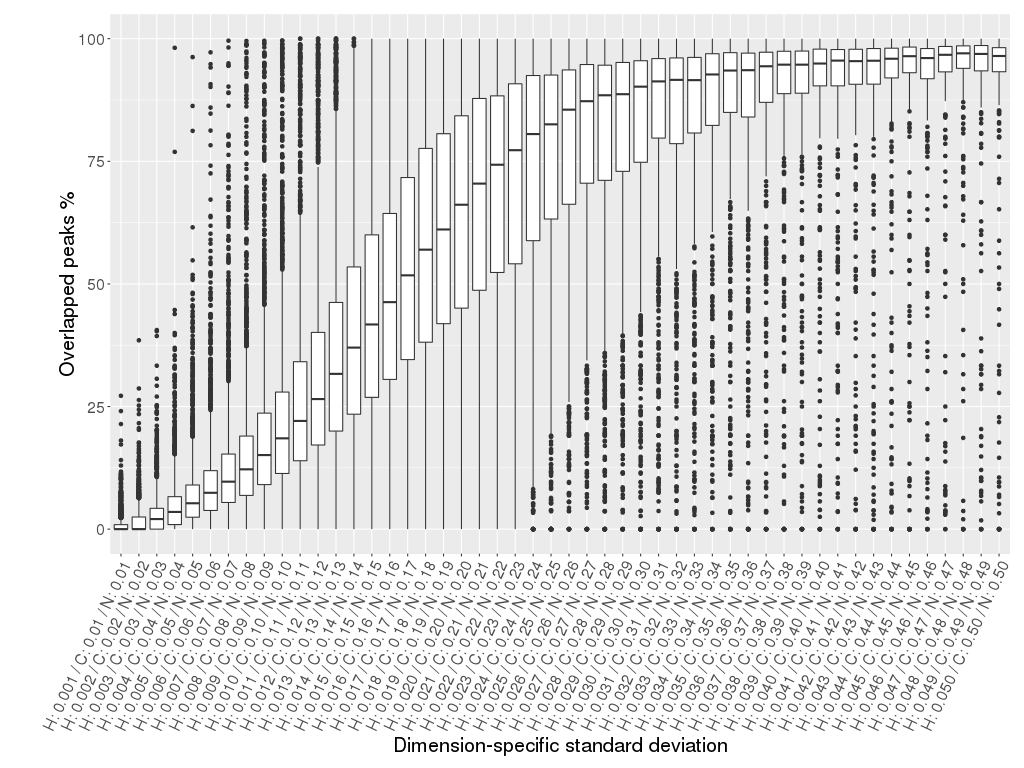


**Figure S5**. Single source of variance in all dimensions: percentage of overlapped peaks with increase in standard deviation values of peak dimensions.


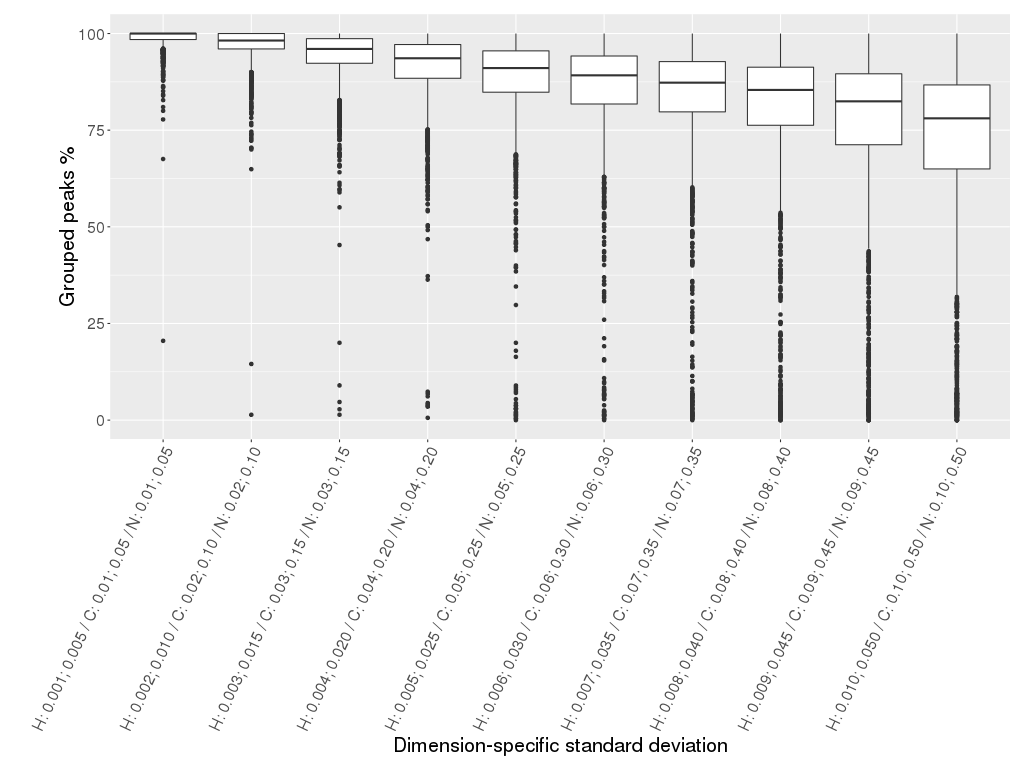


**Figure S6**. Two sources of variance in all dimensions: percentage of grouped (non-overlapped) peaks with increase in standard deviation values of peak dimensions, 20% of peaks have five times larger variance than the remaining 80% of peaks in all dimensions.


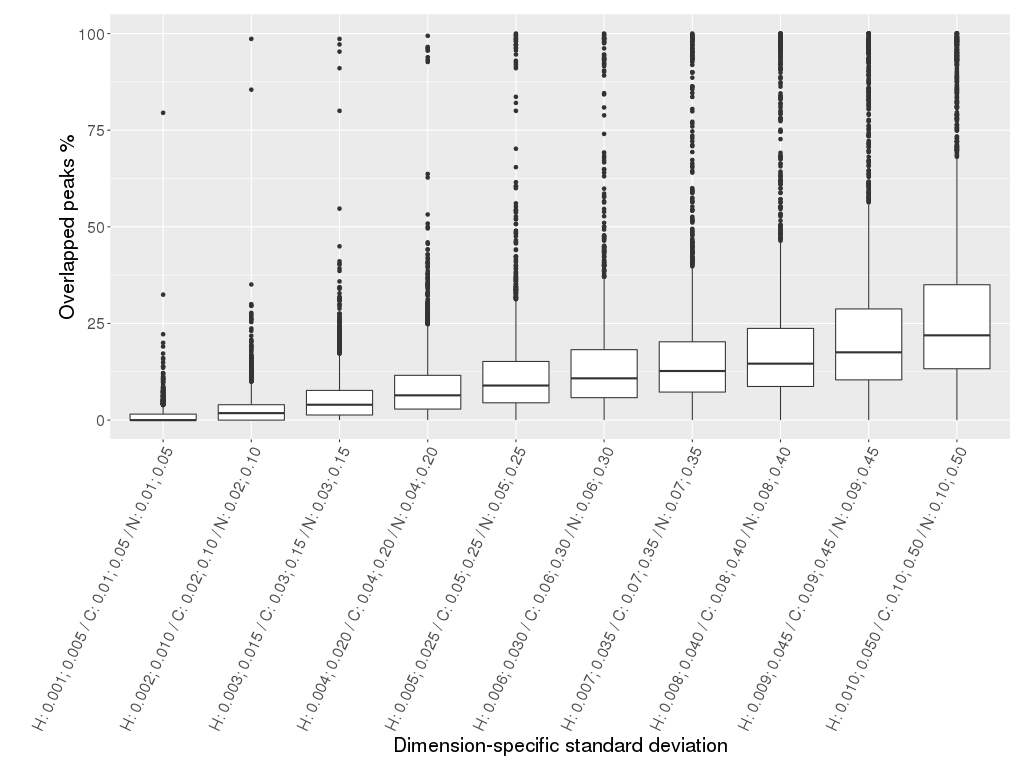


**Figure S7**. Two sources of variance in all dimensions: percentage of overlapped peaks with increase in standard deviation values of peak dimensions, 20% of peaks have five times larger variance than the remaining 80% of peaks in all dimensions.


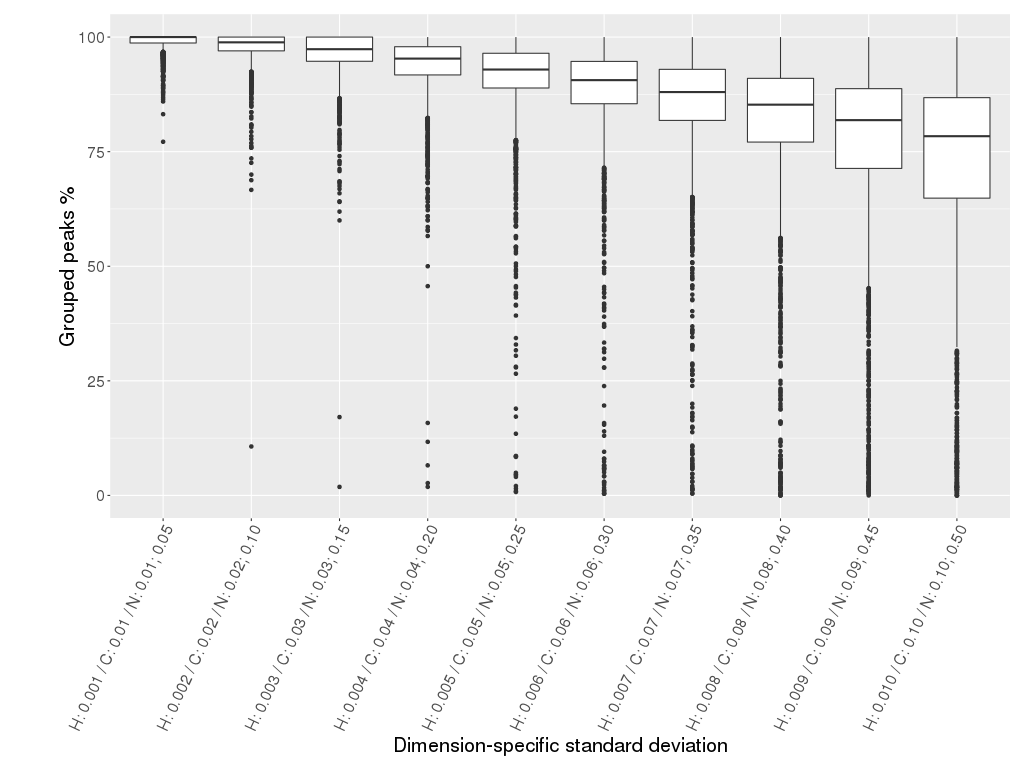


**Figure S8**. Two sources of variance in one dimension: percentage of grouped (non-overlapped) peaks with increase in standard deviation values of peak dimensions, 20% of peaks have five times larger variance than the remaining 80% of peaks in N dimension.


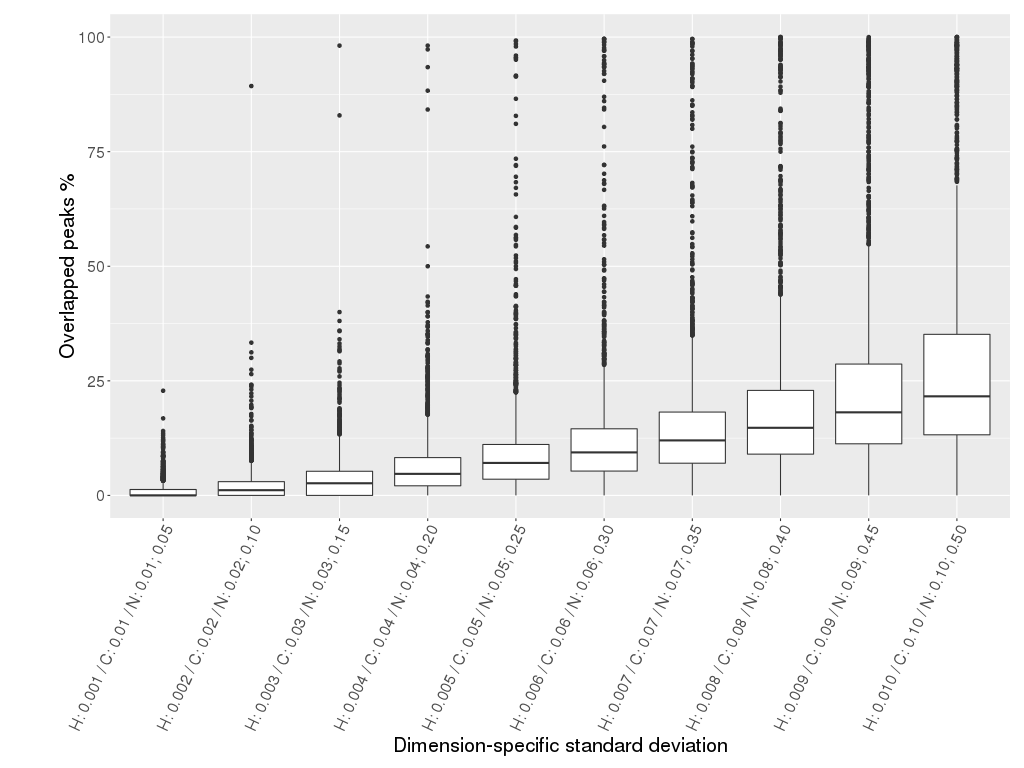


**Figure S9**. Two sources of variance in one dimension: percentage of overlapped peaks with increase in standard deviation values of peak dimensions, 20% of peaks have five times larger variance than the remaining 80% of peaks in N dimension.
