# Supplementary material for: Detecting and accounting for multiple sources of positional variance in peak list registration analysis and spin system grouping
Source: J Biomol NMR. 2017 Aug 16;68(4):281–96. doi: 10.1007/s10858-017-0126-5 (PMC5587626; doi:10.1007/s10858-017-0126-5)
Supplement: Supplementary file 2 — Supplementary material 2 (PDF 174 KB) [file 10858_2017_126_MOESM2_ESM.pdf]

Solution NMR experimental peak lists

Table S1a. *Spin system grouping results for solution NMR derived peak lists using combined registration analysis and grouping algorithm.*

$$\chi^2_{df}(i,j,m,n) = \sum_{l=0}^{df}\left(\frac{(input\ peak\ list_i[l]-input\ peak\ list_m[l])-(root\ peak\ list_j[l]-root\ peak\ list_n[l])}{std[l]\cdot 2}\right)^2$$

Protein / Peak list	Expected peaks	Observed peaks	Ungrouped peaks	Expected spin systems	Identified spin systems	Missing spin systems	Overlapped spin systems	Split spin systems
BPTI / HN(CO)CACB	101	134	17	47	54	0	0	2
CSP / HN(CO)CACB	125	145	39	57	53	12	0	0
ER14 / HN(CO)CACB	194	181	7	93	87	8	2	0
FGF / HN(CO)CACB	273	303	24	128	139	13	2	1
JR19 / HN(CO)CACB	151	141	7	71	67	4	0	0
NS1 / HN(CO)CACB	137	203	36	66	81	26	8	2
RnaseC6572S / HN(CO)CACB	235	282	16	116	130	18	4	2
RnaseWT / HN(CO)CACB	235	403	19	116	181	9	2	1
ZDOM / HN(CO)CACB	134	153	29	67	55	15	3	5
ZR18 / HN(CO)CACB	172	163	3	85	80	5	0	0

Table S1b. *Spin system grouping results for solution NMR derived peak lists using combined registration analysis and grouping algorithm.*

$$\chi^2_{df}(i,j,m,n) = \sum_{l=0}^{df}\left(\frac{(input\ peak\ list_i[l]-input\ peak\ list_m[l])-(root\ peak\ list_j[l]-root\ peak\ list_n[l])}{std[l]\cdot\sqrt{2}}\right)^2$$

Protein / Peak list	Expected peaks	Observed peaks	Ungrouped peaks	Expected spin systems	Identified spin systems	Missing spin systems	Overlapped spin systems	Split spin systems
BPTI / HN(CO)CACB	101	134	17	47	58	0	0	1
CSP / HN(CO)CACB	125	145	39	57	51	12	2	0
ER14 / HN(CO)CACB	194	181	7	93	87	8	2	0
FGF / HN(CO)CACB	273	303	24	128	139	13	2	1
JR19 / HN(CO)CACB	151	141	7	71	67	4	0	0
NS1 / HN(CO)CACB	137	203	36	66	63	26	0	2
RnaseC6572S / HN(CO)CACB	235	282	16	116	110	18	2	4
RnaseWT / HN(CO)CACB	235	403	19	116	180	9	2	3
ZDOM / HN(CO)CACB	134	153	29	67	57	15	3	5
ZR18 / HN(CO)CACB	172	163	3	85	80	5	0	0

Table S1c. *Spin system grouping results for solution NMR derived peak lists using combined registration analysis and grouping algorithm.*

$$\chi^2_{df}(i,j,m,n) = \sum_{l=0}^{df}\left(\frac{(input\ peak\ list_i[l]-input\ peak\ list_m[l])-(root\ peak\ list_j[l]-root\ peak\ list_n[l])}{std[l]\cdot 2.5}\right)^2$$

Protein / Peak list	Expected peaks	Observed peaks	Ungrouped peaks	Expected spin systems	Identified spin systems	Missing spin systems	Overlapped spin systems	Split spin systems
BPTI / HN(CO)CACB	101	134	17	47	49	0	0	1
CSP / HN(CO)CACB	125	145	39	57	53	12	0	0
ER14 / HN(CO)CACB	194	181	7	93	87	8	2	0
FGF / HN(CO)CACB	273	303	24	128	139	13	2	1
JR19 / HN(CO)CACB	151	141	7	71	67	4	0	0
NS1 / HN(CO)CACB	137	203	36	66	66	26	2	2
RnaseC6572S / HN(CO)CACB	235	282	16	116	129	18	4	2
RnaseWT / HN(CO)CACB	235	403	19	116	178	9	2	0
ZDOM / HN(CO)CACB	134	153	29	67	55	15	3	6
ZR18 / HN(CO)CACB	172	163	3	85	80	5	0	0

Table S1d. *Spin system grouping results for solution NMR derived peak lists using combined registration analysis and grouping algorithm.*

$$\chi^2_{df}(i,j,m,n) = \sum_{l=0}^{df}\left(\frac{(input\ peak\ list_i[l]-input\ peak\ list_m[l])-(root\ peak\ list_j[l]-root\ peak\ list_n[l])}{std[l]\cdot\sqrt{3}}\right)^2$$

Protein / Peak list	Expected peaks	Observed peaks	Ungrouped peaks	Expected spin systems	Identified spin systems	Missing spin systems	Overlapped spin systems	Split spin systems
BPTI / HN(CO)CACB	101	134	17	47	54	0	0	1
CSP / HN(CO)CACB	125	145	39	57	53	12	0	0
ER14 / HN(CO)CACB	194	181	7	93	87	8	2	0
FGF / HN(CO)CACB	273	303	24	128	139	13	2	1
JR19 / HN(CO)CACB	151	141	7	71	67	4	0	0
NS1 / HN(CO)CACB	137	203	36	66	77	26	2	2
RnaseC6572S / HN(CO)CACB	235	282	16	116	129	18	4	2
RnaseWT / HN(CO)CACB	235	403	19	116	178	9	2	3
ZDOM / HN(CO)CACB	134	153	29	67	54	15	2	5
ZR18 / HN(CO)CACB	172	163	3	85	80	5	0	0

Solid-state NMR experimental peak lists

Table S2a. *Spin system grouping results for solid-state NMR derived peak lists using combined registration analysis and grouping algorithm.*

$$\chi^2_{df}(i,j,m,n) = \sum_{l=0}^{df}\left(\frac{(input\ peak\ list_i[l] - input\ peak\ list_m[l]) - (root\ peak\ list_j[l] - root\ peak\ list_n[l])}{std[l]\cdot 2}\right)^2$$

Protein / Peak list	Expected peaks	Observed peaks	Ungrouped peaks	Expected spin systems	Identified spin systems	Missing spin systems	Overlapped spin systems	Split spin systems
GB1 / CANCOCX	268	240	70	55	56	1	6	28
GB1 / NCACX	268	463	62	55	65	0	0	19
GB1 / NCOCX	268	474	16	55	82	0	4	10
DsbB / NCACX	940	215	43	175	47	126	14	1
CapGly / NCACX	410	515	16	88	50	33	25	0
CapGly / NCOCX	410	218	25	88	47	38	32	5

Table S2b. *Spin system grouping results for solid-state NMR derived peak lists using combined registration analysis and grouping algorithm.*

$$\chi^2_{df}(i,j,m,n) = \sum_{l=0}^{df}\left(\frac{(input\ peak\ list_i[l] - input\ peak\ list_m[l]) - (root\ peak\ list_j[l] - root\ peak\ list_n[l])}{std[l]\cdot \sqrt{2}}\right)^2$$

Protein / Peak list	Expected peaks	Observed peaks	Ungrouped peaks	Expected spin systems	Identified spin systems	Missing spin systems	Overlapped spin systems	Split spin systems
GB1 / CANCOCX	268	240	70	55	85	1	11	36
GB1 / NCACX	268	463	62	55	86	0	6	27
GB1 / NCOCX	268	474	16	55	107	0	4	26
DsbB / NCACX	940	215	43	175	75	126	12	15
CapGly / NCACX	410	515	16	88	66	33	27	14
CapGly / NCOCX	410	218	25	88	48	38	23	4

Table S2c. *Spin system grouping results for solid-state NMR derived peak lists using combined registration analysis and grouping algorithm.*

$$\chi^2_{df}(i,j,m,n) = \sum_{l=0}^{df}\left(\frac{(input\ peak\ list_i[l] - input\ peak\ list_m[l]) - (root\ peak\ list_j[l] - root\ peak\ list_n[l])}{std[l]\cdot 2.5}\right)^2$$

Protein / Peak list	Expected peaks	Observed peaks	Ungrouped peaks	Expected spin systems	Identified spin systems	Missing spin systems	Overlapped spin systems	Split spin systems
GB1 / CANCOCX	268	240	70	55	56	1	11	18
GB1 / NCACX	268	463	62	55	65	0	0	13
GB1 / NCOCX	268	474	16	55	67	0	4	10
DsbB / NCACX	940	215	43	175	47	126	14	1
CapGly / NCACX	410	515	16	88	57	33	37	5
CapGly / NCOCX	410	218	25	88	50	38	34	2

Table S2d. *Spin system grouping results for solid-state NMR derived peak lists using combined registration analysis and grouping algorithm.*

$$\chi^2_{df}(i,j,m,n) = \sum_{l=0}^{df}\left(\frac{(input\ peak\ list_i[l] - input\ peak\ list_m[l]) - (root\ peak\ list_j[l] - root\ peak\ list_n[l])}{std[l]\cdot \sqrt{3}}\right)^2$$

Protein / Peak list	Expected peaks	Observed peaks	Ungrouped peaks	Expected spin systems	Identified spin systems	Missing spin systems	Overlapped spin systems	Split spin systems
GB1 / CANCOCX	268	240	70	55	81	1	14	33
GB1 / NCACX	268	463	62	55	70	0	0	20
GB1 / NCOCX	268	474	16	55	98	0	6	22
DsbB / NCACX	940	215	43	175	76	126	13	12
CapGly / NCACX	410	515	16	88	79	33	35	17
CapGly / NCOCX	410	218	25	88	48	38	25	3
